# Maternal, newborn and breast milk concentrations of elexacaftor/tezacaftor/ivacaftor in a F508del heterozygous woman with cystic fibrosis following successful pregnancy

**DOI:** 10.3389/fmed.2023.1274303

**Published:** 2023-12-07

**Authors:** Pietro Ripani, Matteo Mucci, Stefano Pantano, Maria Di Sabatino, Francesca Collini, Giulia Ferri, Mario Romano, Antonio Recchiuti

**Affiliations:** ^1^Unità Operativa Semplice a valenza Dipartimentale Centro Regionale di Riferimento per la Fibrosi Cistica, Teramo, Italy; ^2^Department of Medical, Oral, and Biotechnology Science (DSMOB), Center of Advanced Studies and Technology, Università “G. d’Annunzio”, Chieti, Italy

**Keywords:** novel therapies, pregnancy, drug monitoring, F508del CFTR, CFTR modulator therapy, R334W CFTR, mass spectrometry

## Abstract

With the introduction of elexacaftor/tezacaftor/ivacaftor (ETI), more women with cystic fibrosis (CF) are likely to grow families. Hence, an understanding long-term safety and effects of CFTR modulators on fertile women and children while monitoring their concentrations is crucial. Here, we report on the development of an improved LC–MS/MS methodology to measure ETI concentrations in maternal and child blood and breastmilk, applied in one case of successful pregnancy of a 30-year-old woman with CF (F508del/R334W). We observed that ETI remains stable in breastmilk, is absorbed by the infant and can be detected in child plasma. Our results confirm accumulating evidence of a successful pregnancy in women treated with CFTR modulators without significant side effects on the child and provide valuable analytical procedures suitable for the post-marketing evaluation of CFTR modulators in pregnant and lactating women, as well as in their infants.

## 1 Introduction

Highly effective CFTR modulator therapies, such as the triple combination of elexacaftor/tezacaftor/ivacaftor (ETI), have brought about a transformative treatment for people with cystic fibrosis (CF), significantly improving the quality and length of their lives (1, 2). Consequently, an increasing number of women living with CF are expressing the desire for pregnancy and the number of pregnancies among adults with CF is increasing. ETI and other modulators may enhance female fertility by improving physical and psychological status as well as by normalizing the cervical micro-environment (3). Although teratogenicity has not been observed in animals treated with ETI at doses routinely administered to humans (4) and accruing clinical data show no alarming signals (3), there are though concerns on the effects of chronic exposure of a fetus or an infant who may not have CF to ETI. Given the absence of clear data or guidelines, the decision to discontinue ETI during pregnancy and lactation (increasing the risk of maternal health decline) or to continue it (with uncertain fetal/newborn outcomes) remains a dilemma for both women and caregivers.

Here, we report on the development of a pioneering method for measuring the concentration of these compounds in maternal and newborn biological fluids, which we tested in one case of pregnancy of a patient with CF treated with ETI.

## 2 Results

### 2.1 Case

The proband was diagnosed at 18 years of age with CF-causing mutations (F508del/R334W), pancreatic sufficiency, and 115 mmol/L sweat Cl-concentration. Besides chronic lung colonization by *Staphylococcus aureus*, she had atopy and cholestasis requiring treatment with ursodeoxycholic acid (450 mg/die). She was treated at the Abruzzo regional CF center in Atri, Teramo (TE), Italy.

At the age of 31, she was prescribed ETI (Elexacaftor 100 mg/die, Tezacaftor, 50 mg/die, Ivacaftor 75 mg/die) in combination with Ivacaftor (150 mg/die) as standard of care (Kaftrio + Kalydeco, Vertex Pharmaceuticals) in association with flunisolide (Fluspiral) 125 μg 1 puff × 2/day and salbutamol 3 puff × 2/day. Anthropometric characteristics of the patient were as follows: weight = 60 kg; height = 163 cm; BMI = 22 kg/m^2^. Prior to ETI + Iva therapy, the sweat Cl-was 82 mmol/L and the respiratory function was moderately reduced, with a forced expiratory volume percent of predicted (FEV1pp) of 61–66, and multiple diffuse chest bronchiectasis and bronchiolectasis were deduced. She had an average of two episodes of hemoptysis/year and five infection reacutizations/year. Ultrasound scan revealed liver within normal limits with mild echogenicity. The mean total score in the Cystic Fibrosis Questionnaire Revised (CFQ-R) was 66. The six-minute walking test (6MWT) was 500 m (normal range 400–700 m).

The proband initiated ETI + Iva therapy in December 2021 had no relevant adverse side effects, with normal blood tests and negative eye exams (carried out to exclude cataract, which is reported as a side effect of ETI + Iva). Normal blood tests included the following evaluations: total blood cell count, glycemia, liver function tests (albumin, total protein, alkaline phosphatase, alanine transaminase, aspartate aminotransferase, gamma-glutamyl transferase, bilirubin, lactate dehydrogenase), prothrombin time, and pancreatic enzymes.

One month post ETI + Iva, sweat Cl-was 89 mEq/L and FEV1pp raised up to 83. Vital parameters were normal. Body weight was 62 kg, height 163 cm, BMI 23.3, CFQ-R 87, 6MWT 600 m, blood pressure (BP) 116/64 mmHg.

The proband reported had unprotected sexual intercourse with her partner for 9 years without conceiving. However, in March 2022, 3 months after the initiation of ETI + Iva therapy, she announced her pregnancy. Body weight was 64 kg, BMI 24, CFQ-R 83, 6MWT 575 m. FEV1pp was 85. Vital parameters were normal (sO_2_ 100%, respiratory rate 88 bpm/r, BP 112/64 mmHg). Urinary pregnancy exams tested positive (serum beta hCG 14,042 mIU/mL). Gynecological examination confirmed the pregnancy, the correct embryo implant, and the presence of the gestational sac. A fibromatous uterus was reported.

Since safety data on ETI + Iva during human pregnancy were limited to few case reports (5, 6) with only a single large study on 45 pregnant American women (7) and the general recommendation was still to avoid the administration of ETI during pregnancy, we discontinued the therapy with ETI + Iva, with disclosed gravidity in accordance with the proband, and continued the general clinical evaluation regarding the patient’s respiratory and infectious state. However, her symptoms started to deteriorate as early as 1 week after discontinuation: pulmonary function declined (FEV1pp 64), cough increased, and she had a reactivation of pulmonary infection at 1 month post discontinuation (treated with cefuroxime 500 mg/every 8 h for 14 days, os). This worsening of symptoms prompted us to reinitiate therapy with ETI + Ivacaftor in May 2022 (12 weeks of gestation). CFQ-R was 74, BP 103/56 mmHg. The patient tested positive for SARS-COV-2 virus prior to the re-initiation of therapy with CFTR modulators, with generic symptoms, fever, and cough. No specific therapies were given. Echograpic exams showed a normal fetus. In July 2022, FEV1pp had raised up to 86, BP was 100/60 mmHg, and weight 63.7 kg. In January 2023, sweat Cl-was 77 mmol/L.

Pregnancy was regular. From May to July 2022, the proband underwent morphometric echography who did not show any fetal abnormality. Amniotic fluid, auxological parameters, and growth percentiles were normal. No gynecological concerns existed. The oral glucose tolerance test was negative, and the fetal growth followed the normal path. A mild hydronephrosis was suspected upon close prenatal fetal sonoanatomy (resolved at birth).

The proband completed the pregnancy regularly at week 37 + 6 by a Cesarean section. She gave birth to a healthy boy of 3.625 kg (50th percentile), 50 cm of body length (75th percentile), and 37 cm of cranial circumference (>97th percentile). Patent foramen ovale was observed for the newborn at birth. APGAR score was 9–10, and sO_2_ was 100%. The newborn underwent total cell count, alanine transaminase, aspartate aminotransferase, and hemogas blood test in addition to eye exam, otoemission exam, clinical exam, and renal ecograph (all negative). Sweet Cl-was 11 mEq/L and genetic screening revealed that the newborn was carrier of one F508del Cftr allele. The mother continued to breastfeed her child while continuing ETI + Iva therapy, and no significant side effects related to ETI + Iva have been observed in the child thus far.

### 2.2 ETI measurements

In order to assess the presence of ETI in both maternal and fetal biological fluids and mother–child passage, given the limited number of reported analytical methods (8), we developed a new rapid, sensitive, and robust quantification methodology using LC/MS–MS.

Fasting EDTA-plasma and breast milk were collected at 8 a.m., 12 h after the last Iva dose (in the evening) and before the morning administration of ETI + Iva.

The baby was 3 months old and was not breastfed 2 h prior to milk and blood collection. The mother was breastfeeding exclusively.

**Figure 1:**
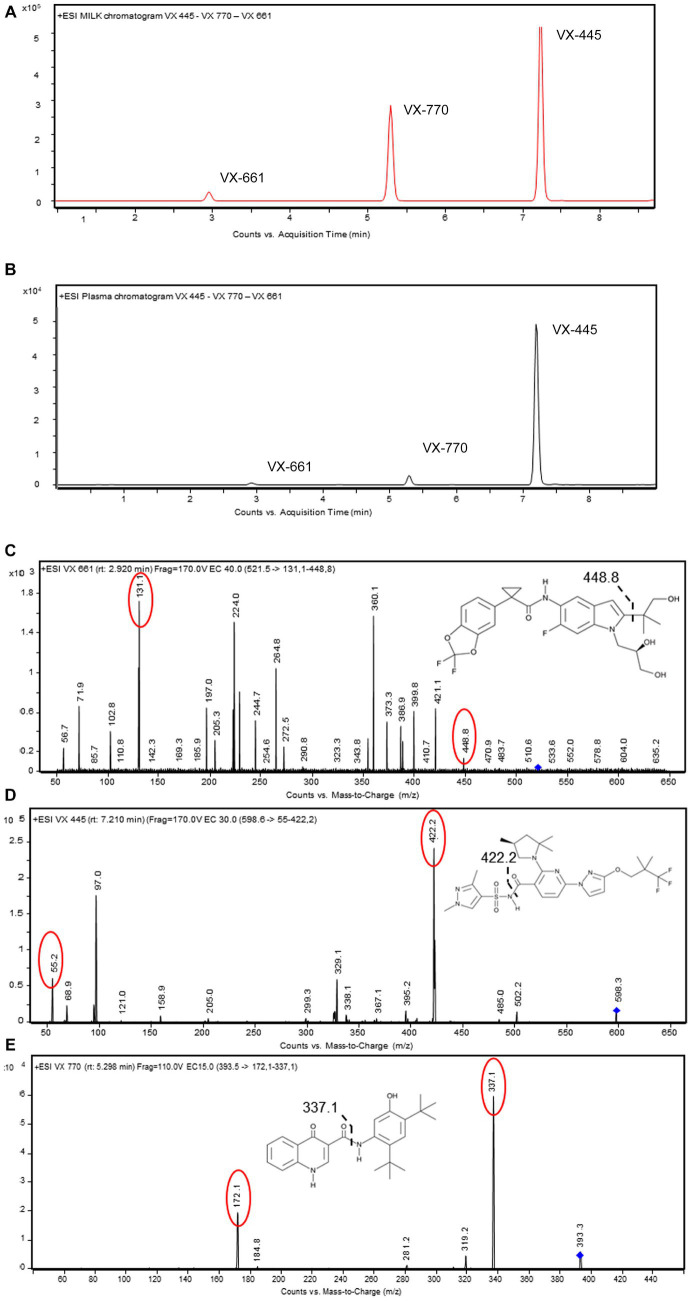
LC–MS/MS method development for measuring ETI in biological samples. **(A,B)** HPLC chromatograms of VX-445, VX-661, and VX-770 spiked (0.05 ng/μL) in pooled blank human plasma or breastmilk. Proteins were removed by precipitation and samples injected (10 μL) into the C18-HPLC system. **(C–E)** Full product ion scan plots of VX-455, VX-661, and VX-770 obtained with MS/MS. Main transitions for each target molecules are in red. Bond cleavage sites are represented by dashed lines. Data are representative from *n* = 20 LC–MS/MS separate injections.

Samples (100 μL) were mixed with four volumes of methanol containing an internal standard (IS), consisting of 10 pg/μL (final concentration) VX 770 d-19 (Cayman Chemical Company), and precipitated by centrifugation (14,000 × g 10 min at 4°C). Ten μL of supernatants were injected into a Ultivo LC–MS–MS system (Agilent Technologies, United States) equipped with LC-1260 Infinity II HPLC (Agilent Technologies, United States). An Agilent Eclipse Plus C18 (50 × 2.1 mm, 1.8 μm) was used with a gradient of water/formic acid/acetonitrile from 55:0.1:45 to 65:0.1:35 vol/vol at 0.3 mL/min flow rate for 8 min of run. The chromatographic separation and MS/MS spectra of each compound are illustrated in Figure 1.

**Figure 2:**
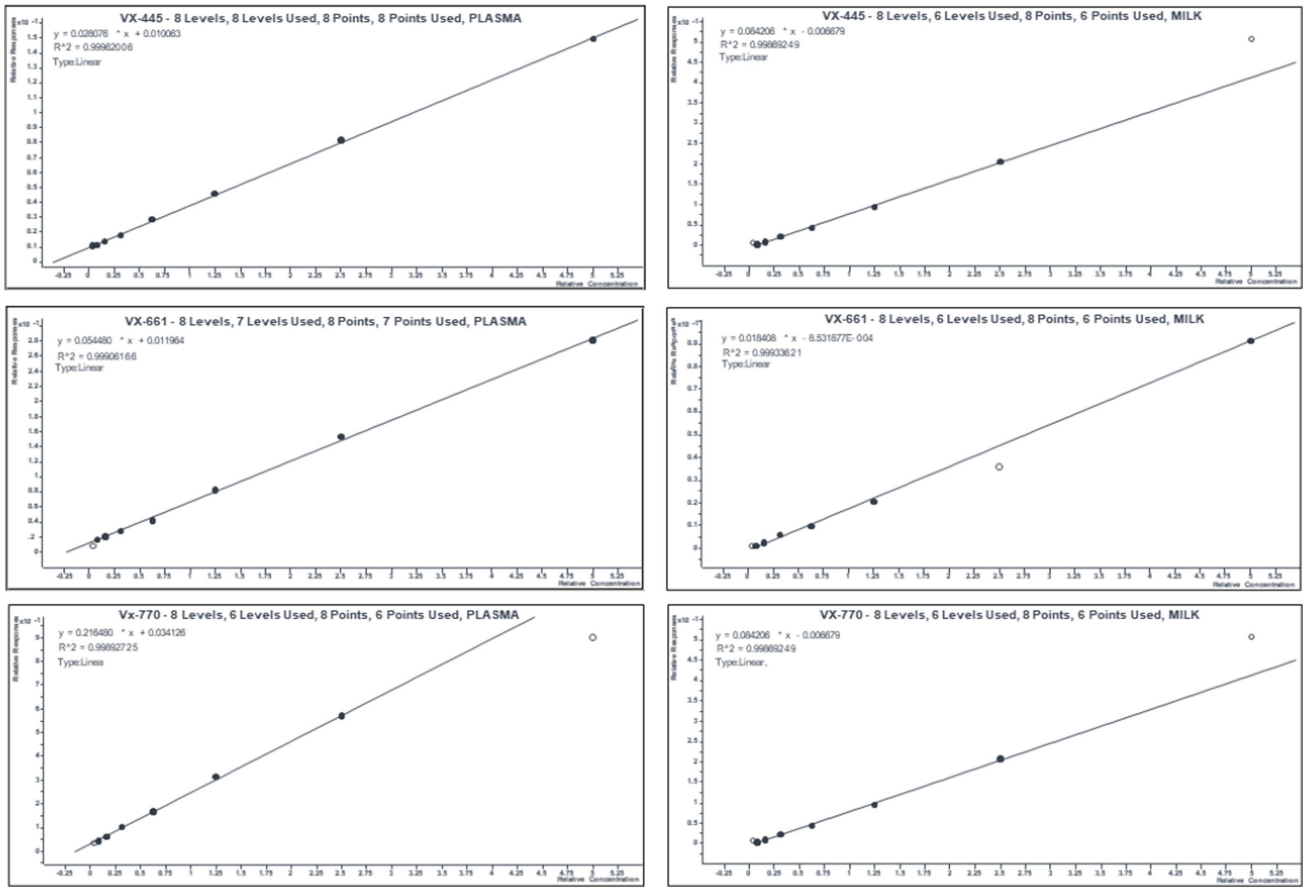
Mean calibration curves of ETI in human plasma and breast milk. Eight-point calibration curves were obtained for each an-analyte with a pool of blank plasma and/ milk spiked with 0.39–50 pg./μL of working solutions of VX-445, VX-661, and VX-770. Relative responses were calculated as ratio between area of the standard (*n* = 5 replicate/concentration) over that of IS.

Standard curves and quality controls were obtained by spiking a pool of blank plasma and/o milk and with analytes from different batches of working solutions of VX-770, VX-661, and VX-445 (from Cayman Chemical Company). The 8-point calibration curve, ranging from 0.39 to 50 pg/μL, which included limit of quantitation, limit of detection, and method detection limit, are shown in Figure 2 and Table 1. Concentrations of VX 661, VX 770, VX 445 in mother’s plasma, breast milk, and child’s plasma are reported in Table 2.

**Table 1:** Analytical performance parameters of the ETI quantification methods.

Name	RT (min)	Transition	Avg. Conc. (ng/mL)	Std. Dev. (ng/mL)	Avg. Conc./SD	MDL (ng/mL)	LOQ (ng/mL)	LOD (ng/mL)	S/N (ng/mL)
**Plasma**									
VX-445	7.279	598.6 → 55.0	5.5450	0.2266	24.67	0.7624	0.6797	2.0345	100.67
VX-661	3.058	521.5 → 449.1	5.2789	0.4159	12.69	1.3995	1.2477	2.6182	∞
VX-770	5.351	393.5 → 172.1	5.6461	0.3112	18.14	1.0471	0.9336	2.5899	∞
**Breastmilk**									
VX-445	7.279	598.6 → 55.0	5.6948	0.1331	42.78	0.4479	1.3311	0.3993	354.03
VX-661	3.058	521.5 → 449.1	5.6850	0.1512	37.59	0.5089	1.5125	0.4537	147.46
VX-770	5.351	393.5 → 172.1	5.3919	0.0648	83.19	0.1944	0.6481	0.1944	402.51

Average concentration, SD, method detection limit (MDL), limit of quantification (LOQ), limit of detection (LOD), and signal-to-noise (SN) were determined in human plasma and breastmilk from 3 separate determinations. MDL, LOQ, and LOD were calculated using 6 replicates of the 0.0625 ng/μL standard.

**Table 2:** Concentrations of VX-661, VX-770, and VX-445 measured in plasma and breastmilk (mean ± SD from *n* = 3 separate determinations).

Drug (ng/mL)	VX-661	VX-445	VX-770
Maternal plasma	1017.57 ± 80.02	1185.2 ± 114.01	1071.5 ± 80.12
Breast milk	761.3 ± 22.62	1049.07 ± 68.77	1794.50 ± 4.95
Child plasma	13.91 ± 2.25	4.66 ± 1.15	23.93 ± 2.95

## 3 Discussion

Here, we report a case of a woman with CF who delivered a healthy, term infant while remaining on ETI therapy for most of her pregnancy. We quantified ETI in breast milk and in the child plasma.

Expression of CFTR in female reproductive organs has been established ~30 years ago by Tizzano and colleagues (9). Although the reproductive anatomy of women with CF is generally similar to that of women without CF, abnormal CFTR function can lead to increased mucus viscosity, pH imbalances, dysbiosis, and chronic inflammation that can reduce fertility. Suboptimal nutrition status and overall health conditions may also decrease the chances of becoming pregnant for some women with CF (10). With the advent of CFTR modulators, fertility of women with CF is expected to increase. Consistent with this, few cases of pregnancies shortly after the initiation of CFTR modulator therapy have been reported, and in phase 3 clinical trials with ivacaftor, ~2% of the participating women disclosed pregnancy during the study. ETI was approved by the Italian Medicine Agency (AIFA), the government’s regulatory authority, for patients with CF older than 12 years with two or a single F508del allele in July 2021 (AIFA DG. 784 and 786 07/1/2021). Hence, our case represents one of the earliest reporting a successful pregnancy in women with CF upon therapy with ETI in Italy. Given that 90% of women with CF eligible for CFTR modulators are in their childbearing age (11, 12), our results demonstrate further the timeliness of properly designed clinical studies aimed at ascertaining the basic effects of CFTR modulators on fertility, which is likely to be a growing desire for people with CF.

Our results show that ETI can pass and remain chemically stable in breast milk for a long time after intake and into infant plasma in high ng/mL ranges, confirming preclinical studies and other reports on Ivacaftor, Ivacaftor/Lumacaftor, and Tezacaftor/Ivacaftor (8), or ETI (5). Even if accruing evidence from sparse case reports and preclinical toxicity studies are reassuring on the safety of CFTR modulators, there have been cases of congenital cataracts in babies exposed to ETI *in utero* and during breast feeding (12). In keeping with other case reports from real-life experience, our results indicate that growth, eye, and blood tests were normal in the child exposed to ETI + Iva *in utero* and after birth.

The fact that CFTR modulators can pass into breast milk and be absorbed by infants can be advantageous for treating children with CF, similarly to the case in which ETI + Iva was used to intentionally treat CF-associated meconium ileus (13). Considering the concentrations measured in breast milk and the average daily intake of milk [~720 mL/day at 1 month, ~750 mL/day at 3 months, ~850 mL/day at 6 months (14)] by healthy babies, the following milk-delivered doses can be estimated: VX-661 ~ 800 μg/day, VX-445 ~ 1,000 μg/day, VX-770 ~ 700 μg/day in the first month of age; VX-661 ~ 830 μg/day, VX-4451100 μg/day, VX-770 ~ 740 μg/day at 3 months; VX-661 ~ 940 μg/day, VX-4451270 μg/day, VX-770 ~ 830 μg/day at 6 months. These data deserve clinical consideration. On the contrary, further medical aspects should be considered in the case of heterozygous children, for whom the risks of long-term side effects of CFTR modulators may outweigh the benefits associated with improved CFTR function. These aspects impose an accurate clinical monitoring of the baby’s health over the breastfeeding period. The different concentrations of VX-661 between maternal plasma and breast milk could be explained by physiologic changes in pregnancy that may alter the pharmacokinetics of this molecule. Additionally, increases in maternal body fat during pregnancy can change VX-661 distribution between plasma and breast milk. Moreover, VX-661 may undergo a more rapid metabolism compared to other compounds. Furthermore, the different ratio or percentage between child plasma and breastmilk concentrations for the three molecules could depend upon solubility and pharmacokinetics aspects.

Further studies on ETI pharmacokinetics in pregnancy will be useful to clarify these points.

The methodology, developed and employed here, represents a step forward over previous published methods (15, 16) since it allows quantification of ETI from a small amount of biological fluids, thus minimizing the impact of withdrawal procedures. It also has a linear dynamic range of pg./mL [1,000 times lower than the one reported in (16)], which enables a more precise and sensitive ETI monitoring. A detailed description of the methodology will be published separately. Thus, the present procedure can be useful for post-marketing evaluation of CFTR modulators in patients with CF, including pregnant/lactating women and their babies. At the time this report was released, the Maternal and Fetal Outcomes in the Era of Modulators (MAYFLOWER) study sponsored by the CF Foundation and CF Therapeutics Development Network is ongoing (17).

Our work has some limitations: it reports a single case and lacks a study of ETI kinetics following administration.

In conclusion, here we present one of the few cases registered in Italy of successful delivery upon therapy with ETI without adverse pregnancy outcomes and post birth abnormalities, thus contributing to the continuous data on the use of ETI in pregnant and breast-feeding women, which adds a crucial wealth of knowledge in the absence of large clinical studies. We also present the development and use of a LC–MS/MS method for quantifying ETI in human biological fluids, which can be valuable in clinical laboratories for directing medical decisions.

## 4 Patient perspective

From the patient and CF community perspective, this case highlights that spontaneous conception and pregnancy are possible in female with CF treated with ETI, and urges more women to participate in a large clinical trial to better understand the long-term effects of ETI on the health of the newborn.

## Data availability statement

The datasets presented in this article are not readily available because of ethical/privacy restrictions. Requests to access the datasets should be directed to the corresponding authors.

## Ethics statement

Written informed consent was obtained from the individual for the publication of any potentially identifiable images or data included in this article.

## Author contributions

PR: Data curation, Supervision, Writing – original draft, Investigation, Validation. MM: Data curation, Investigation, Methodology, Writing – original draft, Writing – review & editing. SP: Investigation, Writing – original draft. MD: Investigation, Writing – original draft. FC: Investigation, Writing – original draft. GF: Investigation, Writing – original draft. MR: Writing – original draft. AR: Writing – original draft, Conceptualization, Data curation, Funding acquisition, Supervision, Writing – review & editing.
